# The Isoforms of Estrogen Receptor Alpha and Beta in Thyroid Cancer

**DOI:** 10.3389/fonc.2022.916804

**Published:** 2022-06-24

**Authors:** Zhongqin Gong, Shucai Yang, Minghui Wei, Alexander C. Vlantis, Jason Y. K. Chan, C. Andrew van Hasselt, Dongcai Li, Xianhai Zeng, Lingbin Xue, Michael C. F. Tong, George G. Chen

**Affiliations:** ^1^ Department of Otorhinolaryngology, Head and Neck Surgery, Prince of Wales Hospital, The Chinese University of Hong Kong, Hong Kong, Hong Kong SAR, China; ^2^ Department of Clinical Laboratory, Pingshan District People’s Hospital of Shenzhen, Shenzhen, China; ^3^ Department of Head & Neck Surgery, Cancer Hospital Chinese Academy of Medical Sciences, Shenzhen Center, Shenzhen, China; ^4^ Shenzhen Key Laboratory of Ear Nose Throat (ENT), Institute of ENT & Longgang ENT Hospital, Shenzhen, China

**Keywords:** ERα, ERβ, isoforms, splicing, thyroid cancer

## Abstract

The incidence of thyroid cancer was predominant in women, indicating that the sex hormone may have a role in thyroid cancer development. Generally, the sex hormone exerts its function by binding to the correspondent nuclear receptors. Therefore, aberrant of these receptors may be involved in the development of thyroid cancer. Estrogen receptor alpha (ERα) and beta (ERβ), two main estrogen receptors, have been reported to have an important role in the pathogenesis of thyroid cancer. When the ERα and ERβ genes undergo the alternative RNA splicing, some ERα and ERβ isoforms with incomplete functional domains may be formed. To date, several isoforms of ERα and ERβ have been identified. However, their expression and roles in thyroid cancer are far from clear. In this review, we summarized the expressions and roles of ERα and ERβ isoforms in thyroid cancer, aiming to provide the perspective of modulating the alternative RNA splicing of ERα and ERβ against thyroid cancer.

**Graphical Abstract:**
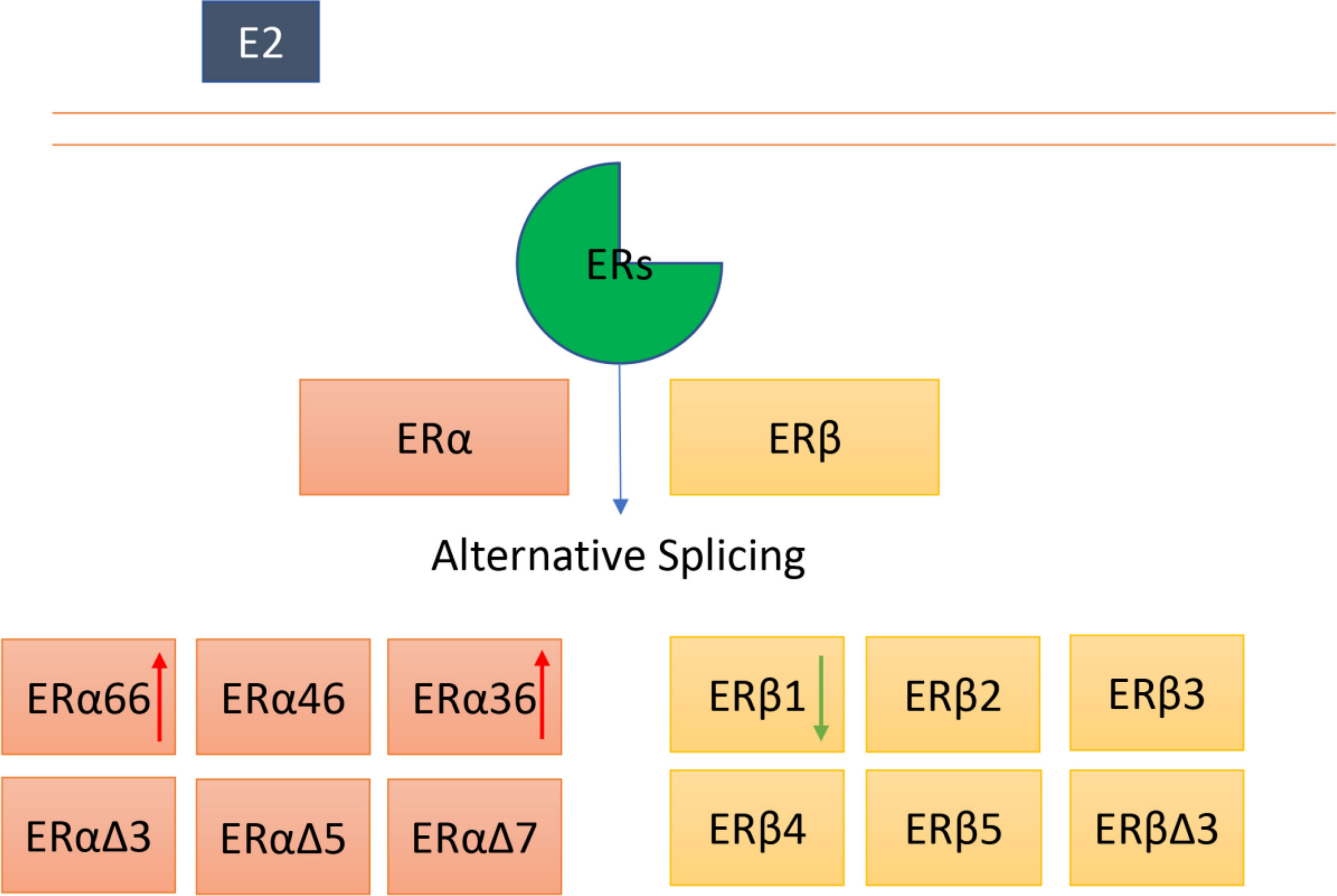
ERα and ERβ can undergo alternative splicing, and several isoforms of ERα and Erβ have been identified. The roles of ERα and ERβ full length in thyroid cancer appear to be controversial. Moreover, the exact functions of other isoforms in thyroid cancer remain largely unknown. The red arrow indicates an increased expression, the green arrow represents a deceased expression in thyroid cancer.

## Introduction

The morbidity of thyroid cancer has been rapidly increased during the past decades (1, 2). The increased rate in women was particularly pronounced (2–5). The biased occurrence of thyroid cancer between males and females suggests that the sex hormone may play a central role in the initiation of thyroid cancers or certain types of thyroid cancers. Traditionally, estrogen is the primary female sex hormone mainly responsible for the control of functions of the female reproductive system. In the genomic pathway, estrogen exerts its physiological functions by binding to specific nuclear receptors, the estrogen receptors (ERs), which activate transcriptional processes and/or signaling events and thus control the gene expression (6). ERs can express in both male and female organs/tissues. Therefore, the ERs are critical in the maintenance of health.

Numerous studies have shown that the critical and opposite roles of ERα and ERβ in the development and progression of thyroid cancer. For example, Maura Di Vito et al. reported that the mRNA and protein of ERα, but not ERβ, was upregulated in thyroid cancer, suggesting that ERα has a vital role in thyroid cancer (7). In addition, ERα positive and ERβ negative were associated with a more aggressive phenotype of T1 and T2 thyroid cancer (8, 9). Estrogen induced the metastatic potential of thyroid cancer through ERα and ERβ (10). Yanhong Huang et al. evaluated the expression of ERα and ERβ by immunohistochemical staining, and they reported that estrogen-activated ERα might mediate the stimulatory effect on thyroid cancer growth and progression (11). However, ERβ was negatively correlated with mutant P53, suggesting that ERβ has some inhibitory actions in thyroid cancer (11). ERα is significantly correlated with distant metastases and poorly differentiated thyroid cancer with multicentricity cases, whereas ERβ is significantly associated with lymph node metastases in follicular thyroid cancer (12). These studies have all suggested that ERα and ERβ play an important role in thyroid cancer.

Our previous studies have also illustrated the significance of ERs in thyroid cancer. We found crosstalk between ERs and peroxisome proliferator-activated receptor gamma (PPAR-γ). The interaction between PPAR-γ and ERβ inhibited the proliferation and migration of thyroid cancer (13). ERα induced prosurvival autophagy through generating the reactive oxygen species and activating ERK1/2 in thyroid cancer (14). In addition, we also reported that the differential role of ERα and ERβ in thyroid cancer mediated the production of endogenous PPAR-γ ligand (15). Upregulated ERα/ERβ ratio by PES1 will promote the occurrence and development of papillary thyroid cancer (PTC) (16).

The roles of ERα and ERβ in thyroid cancer appear to be convincing, and the signal pathway of estrogen and estrogen receptors in the development of thyroid cancer has been well reviewed (17). However, there is a controversial result showing the association of the expression of ERα with a good outcome in thyroid cancer. Giacomo sturniolo et al. (18) evaluated the expression of ERα in 203 PTC, and they observed an association between ERα expression and a favorable outcome in their cohort. The cause of such a controversial result remains unknown.

However, it is possible that controversial results are related to ERα antibodies used. For example, the antibody used by Giacomo sturniolo and colleagues is ERα SP1 clone, which was a synthetic peptide derived from the C-terminal of human estrogen receptor (18). The antibody used by Yanhong Huang and colleagues is ERα 1D5 clone, which is a recombinant human estrogen receptor protein (11). The antibodies recognized different regions of ERα protein might result in different expression patterns since several isoforms of ERα have been identified.

The signaling mechanism of ERs and their expression and roles in thyroid cancer have been well-reviewed (6, 17, 19, 20). Therefore, this review focused on the alternative splicing of ERs or isoforms of ERα and ERβ in thyroid cancer (**Table 1**).

**Table 1:** The expression of ERs isoforms and their roles in thyroid cancer.

ER isoforms	Relative level	Role	Effects	Reference
ERα66 (ERα)	High	Oncogenic	Correlate to aggressive phenotype	(7–9)
ERα66	High	Inhibitory	Correlate to favorabel outcome	(18)
ERα46	N.A	N.A	N.A	N.A
ERα36	High	Oncogenic	Promote proliferation and invasion	(21)
ERαΔ3	N.A	N.A	N.A	N.A
ERαΔ5	N.A	N.A	N.A	N.A
ERαΔ7	N.A	N.A	N.A	N.A
ERβ	Low	Inhibitory	Negatively correlate with mutant P53	(11)
ERβ	N.A	Oncogenic	Correlated to lymph node metastsis	(12)
ERβ	N.A	Oncogenic	Promote cancer-stem like properties	(22)
ERβ2	N.A	Oncogenic	Associate with the progression	(23)
ERβ3	N.A	N.A	N.A	N.A
ERβ4	N.A	N.A	N.A	N.A
ERβ5	N.A	N.A	N.A	N.A
ERβΔ3	N.A	N.A	N.A	N.A

NA, not available.

## Alternative Splicing and Thyroid Cancer

Alternative splicing of protein-coding mRNAs is an essential regulatory mechanism in eukaryotic gene expression that controls the proper function of proteins. The alternative is a fundamental biological process that allows for considerable proteomics diversity and complexity from the limited approximately 20,000 genes (24). However, aberrant alternative splicing may lead to cancer development, and understanding aberrant alternative splicing can facilitate cancer diagnosis and therapy (25, 26). Overall, The abnormal regulation of alternative splicing that can produce multiple different isoforms and diversify protein expression may lead to development of tumors.

Alternative splicing events frequently occur in thyroid cancer. Zenghong Wu et al. found that 45150 alternative splicing events in 10446 thyroid cancer cells derived from 506 patients (27). Furthermore, they found that the alternative splicing signatures were significantly associated with thyroid cancer patients’ overall survival (27). Baoai Han et al. showed that abnormal alternative splicing events might play critical roles in the development and progression of thyroid cancer by participating in changes in molecular structure, homeostasis of the cell environment (28). To date, several isoforms of ERα and ERβ have been reported, given the significance of alternative splicing and ERs isoforms in thyroid cancer, indicating that the expression and role of ERα and ERβ isoforms in thyroid cancer are important. Therefore, in the following section, we would discuss the ERα and ERβ isoforms in thyroid cancer.

## ERα and Its Isoforms in Thyroid Cancer

According to the national center for biotechnology information database (https://www.ncbi.nlm.nih.gov/), ERα is located in 6q25.1-q25.2. The ERα protein contains an N-terminal ligand-independent transactivation domain, a central DNA binding domain, a hinge domain, and a C-terminal ligand-dependent transactivation domain. The N-terminal ligand-independent transactivation domain encompassed a ligand-independent activation function (AF1) domain involved in the transcriptional activation of target genes. The DNA binding domain mediates sequence-specific binding of ERs to DNA sequences in the target gene denoted estrogen-responsive elements (EREs). The C-terminal ligand-dependent transactivation domain contains a ligand-dependent activation domain (AF2) (29, 30). The protein localizes to the nucleus, where it may form either a homodimer or a heterodimer with ERβ.

**Figure 1:**
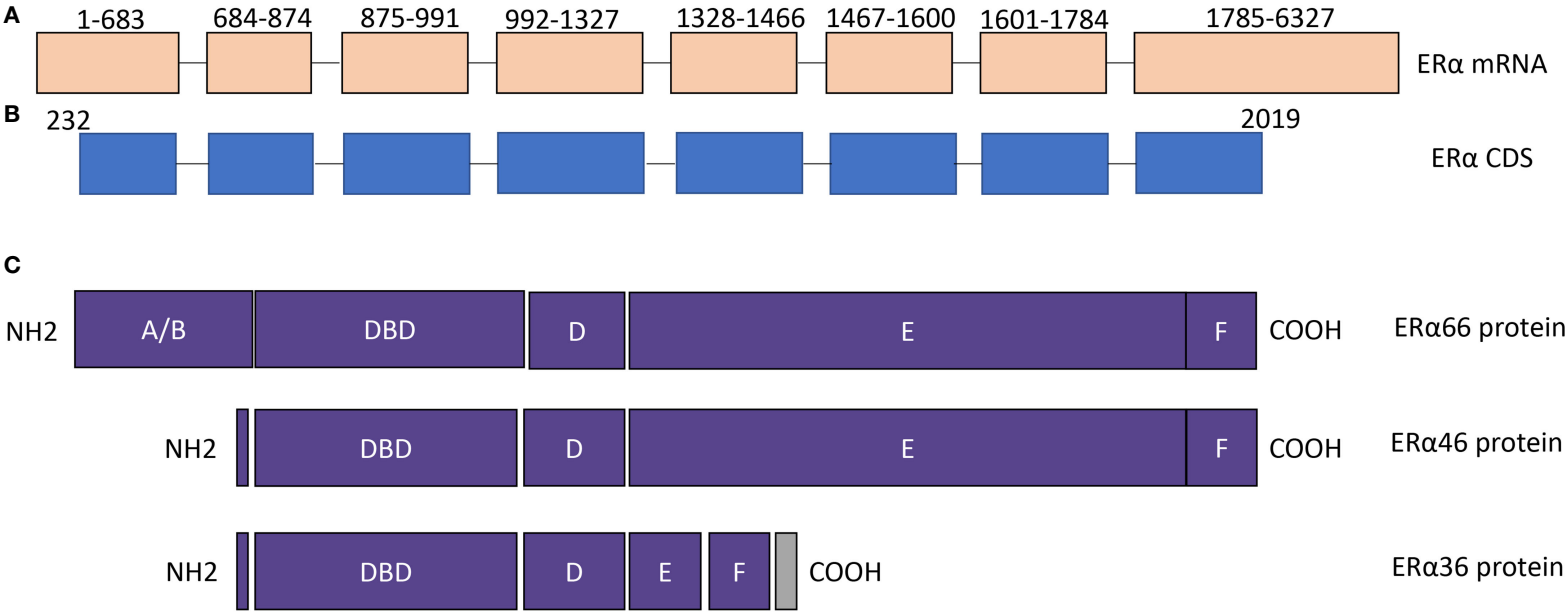
The structure of ERα isoforms. **(A)** ERα mRNA contains 8 exons. NCBI reference sequence: NM_000125.4, transcript variant 1. **(B)** ERα coding region. **(C)** The protein structure of ERα66, ERα46, and ERα36. The difference among ERα66, ERα 46, and ERα36 is mainly located in AF1 and AF2 domains. DBD, DNA binding domain.

Several alternative splicing isoforms of ERα have been identified, including ERα wild type/full length (ERα66), ERα46, and ERα36 (**Figure 1**). The isoforms of ERα have incomplete function domains that may alter their roles in thyroid cancer. The expression and role of ERα66 in thyroid cancer have been described in the previous section. Therefore, this section would focus on the ERα46, ERα36 and exon-deleted ERα isoforms.

### ERα46 and Thyroid Cancer

ERα46 was first identified and characterized in osteoblasts (31). ERα46 is generated by alternative splicing of an ERα66 gene product, which results in exon 1 being skipped with a start codon in exon 2 used to initiate translation of the protein. Consequently, compared to ERα66, the ERα46 protein lacks amino acids 1-173, which codes N-terminal ligand-independent transactivation domain (AF1). Therefore, ERα46 has an incomplete AF1 domain.

Functional analysis revealed that ERα46 could heterodimerize with the ERα66 as well as the ERβ (31). However, the expression and role of ERα46 in thyroid cancer remain largely unknown. In another ER-related cancer, breast cancer, the expression of ERα46 was observed in over 70% of breast tumors among 116 ERα66 positive human breast tumors (32). In addition, ERα46 decreased the proliferation rate of breast cancer MCF7 cells in response to 17β estradiol (32). The data suggested that ERα46 inhibited tumor cell functions, which is different from ERα66.

Furthermore, the reduced expression of ERα46 was found in tamoxifen-resistant breast cancer cells, and the force overexpression of ERα46 in these tamoxifen-resistant breast cancer cells restored growth inhibition by tamoxifen (33). A study reported that the enhanced expression of ERα in breast cancer was associated with thyroid cancer occurrence, suggesting that ERα may have a role in the link between breast cancer and thyroid cancer (34). However, no data shows the expression and the role of ERα46 in thyroid cancer. Further studies are necessary.

### ERα36 and Thyroid Cancer

ERα36 isofrom is shorter than ERα46. ERα36 lacks both AF-1 and C-terminal ligand-dependent transactivation domain (AF2), and the last 138 amino acids are replaced with a unique 22 amino acid sequence. It was first identified and cloned by Zhaoyi Wang and colleagues, and ERα36 is predicted to function as a dominant-negative effector of ERα66 mediated estrogen-responsive gene pathways and has the potential to trigger membrane-initiated mitogenic estrogen signaling (35, 36). Structurally, ERα36 has an incomplete AF1 domain and an AF2 domain. Therefore, understanding the role of ERα36 in thyroid cancer is vital for us to develop ERs as therapeutic targets.

There are limited studies on the ERα36 in thyroid cancer. The expression of ERα36 proteins was analyzed in 218 primary PTC by immunohistochemistry staining and it was found that its expression was upregulated in thyroid cancer (21). The functional study showed that upregulation of ERα36 by E2 enhanced the proliferation, invasion, and migration of PTC cells. The results suggested that increased expression of ERα36 is associated with aggressive thyroid cancer (21). Given the significance of ERα36 in cancer development and progression (37), further investigation of ERα36 in thyroid cancer may provide us with novel insight into the pathogenesis of thyroid cancer.

### Exon-Deleted ERα Isoforms

In addition to ERα46 and ERα36, several exon-deleted ERα isoforms have been reported in breast cancer, such as exon 3 deleted ERα (ERαΔ3), exon 5 deleted ERα (ERαΔ5), exon 7 deleted ERα (ERαΔ7) (38). As shown in **Figure 1**, exon 3 codes for the DNA binding domain, exon 5 and exon 7 codes for part of the AF2 domain. Therefore, each exon-deleted ERα isoform may alter the function of ERα due to the alteration in functional domains, and their roles in thyroid cancer is warrant further studying.

To date, the expression and function of ERα isoforms in thyroid cancer were far from clear. Further studies were warranted to investigate it.

## ERβ and Its Isoforms in Thyroid Cancer

According to the national center for biotechnology information database (https://www.ncbi.nlm.nih.gov/), the ERβ gene is located at 14q23.2-q23.3. The ERβ protein contains an N-terminal ligand-binding domain, DNA binding domain, and C-terminal ligand-binding domain. ERβ is classified as the nuclear receptor, and mainly located in the nucleus. However, The expression of ERβ also can be observed in the cytoplasm and mitochondrial (20, 39). The impact of subcellular localization on the ERβ function remains unclear.

Structurally, there is only a 16% similarity between the N-terminal ligand-binding domain of ERα and ERβ. In contrast, the DNA binding domain is highly conserved between ERα and ERβ with 97% amino acid identity. The C-terminal ligand-binding domains of ERα and ERβ show a 59% overall amino acid sequence identity (29).

Generally, the function of ERβ is opposite to ERα and it may act as a tumor suppressor in thyroid cancer (40). Downregulation of ERβ will decrease its inhibitory role in thyroid cancer. Our previous study has found that the methylation of the ERβ 5’-untranslated region will attenuate its inhibitory effect on ERα gene transcription and promote the initiation and progression of PTC (41). However, controversial results reported that the expression of ERβ was upregulated by lncRNA-H19 to promote cancer stem-like properties in thyroid cancer, suggesting that ERβ may exert its oncogenic role in thyroid cancer (22).

**Figure 2:**
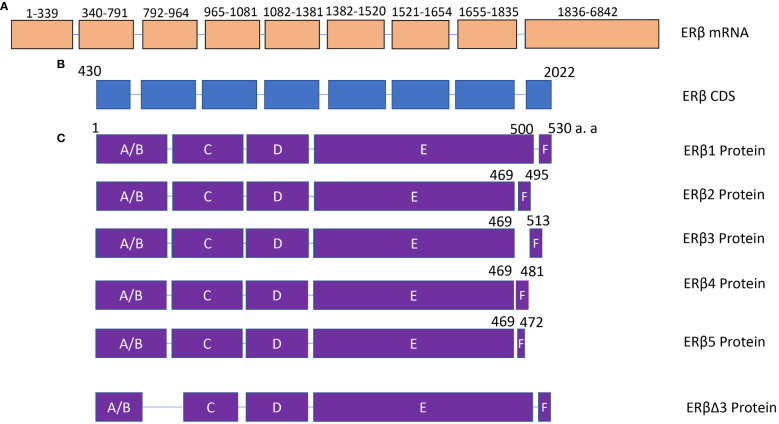
The structure of ERβ isoforms. **(A)** ERβ mRNA contains 9 exons. NCBI reference sequence: NM_001437.3, transcript variant a. **(B)** ERβ coding region. **(C)** The protein structure of ERβ1, ERβ2, ERβ3, ERβ4, ERβ5 and ERβΔ3. The difference among ERβ1, ERβ2, ERβ3, ERβ4, and ERβ5 is mainly located in the C-terminal domain. DBD, DNA binding domain.

Similar to ERα, several isoforms of ERβ have been identified in human cells. In 1998, 5 isoforms of ERβ were cloned and characterized, and named from ERβ1 (Erβ full length) to ERβ5. All these five ERβ isoforms have novel C-terminus (42). Another splicing isoform of ERβ was identified in 2001, and exon 3 was deleted from ERβ, named ERβΔ3 (43). Missing exon 3 altered the subnuclear localization and capacity for transcriptional activation (43). Therefore, alternative splicing will change the function domain of ERβ (**Figure 2**), subsequently affecting its function in thyroid cancer. This section would discuss the expression and roles of ERβ and its isoforms in thyroid cancer.

Though several isoforms of ERβ have been identified for many years, limited studies have been performed to analyze ERβ isoforms in thyroid cancer. Wenwu Dong et al. (23) evaluated the expression of ERβ2 in 106 PTC tissues. They reported that the expression of ERβ2 was positively associated with Ki-67 expression in female patients with advanced reproductive age (>45 years, in low-estrogen status) and with VEGF expression in male PTC patients with reproductive age (18~45 years, in low-estrogen status) (P=0.005 and P=0.044, respectively). There was no association between ERβ2 expression and tumor size, extrathyroidal extension, and tumor-node-metastasis stage in PTC patients. In addition, the expression of ERβ2 was lower in female patients of reproductive age (18~45 years, in relatively high-estrogen status) with lymph node metastasis than in those patients without lymph node metastasis (P=0.035). The results suggested that the expression of ERβ2 in PTC is associated with the progression of the disease (23).

Overall, the role of ERβ in cancer is important. It has been proposed as a promising marker and potential target in cancer metastases (44). ERβ was also correlated with the tumor microenvironment (45). However, the expression and roles of ERβ isoforms remain largely unknown.

The functional domains of ERs will respond to different modulators and degraders (46, 47). Modulations of different ERs domains may have therapeutic impacts (48). The alternative splicing of ERs can result in an incomplete domain, thus affecting the treatment’s efficiency. Therefore, the investigation should focus on the isoforms of ERs in thyroid cancer.

## Conclusion and Perspective

The ERα and ERβ in thyroid cancer are multifaced and complicated. This review has focused on the ERα and ERβ isoforms in thyroid cancer. Given the significance of ERα and ERβ in the development of thyroid cancer and the perspective potential of estrogen receptor modulators and degraders in the treatment of thyroid cancer, the investigation of ERα and ERβ isoforms in the development and progression of thyroid cancer will provide us with a new avenue for the understanding and treatment of thyroid cancer.

## Author Contributions

All authors listed have made a substantial, direct, and intellectual contribution to the work, and approved it for publication.

## Funding

This study was supported by grants from the National Natural Science Foundation of China (No.81972493), and the Research Grants Council of the Hong Kong Special Administrative Region (CUHK 14108921).

## Conflict of Interest

The authors declare that the research was conducted in the absence of any commercial or financial relationships that could be construed as a potential conflict of interest.

## Publisher’s Note

All claims expressed in this article are solely those of the authors and do not necessarily represent those of their affiliated organizations, or those of the publisher, the editors and the reviewers. Any product that may be evaluated in this article, or claim that may be made by its manufacturer, is not guaranteed or endorsed by the publisher.
